# *Salmonella enterica serovar Typhimurium-*induced alterations in inflammatory chemokine mRNA expressions and hemato-biochemical variations in three different strains of chicken

**DOI:** 10.3389/fgene.2025.1645142

**Published:** 2026-01-07

**Authors:** Shabir Mir, Nazir A. Ganai, Syed M. Ahmad, Ishraq Hussain, Gowhar G. Sheikh, Nusrat Nabi, Hassan A. Rudayni, Ahmed A. Allam

**Affiliations:** 1 Division of Animal Genetics and Breeding, Faculty of Veterinary Sciences and Animal Husbandry (FVSc & AH), SKUAST-K Srinagar J&K INDIA, Srinagar, India; 2 FVSc and AH Shuhama SKUAST-K Srinagar, Srinagar, India; 3 Department of Biology, College of Science, Imam Mohammad Ibn Saud Islamic University (IMSIU), Riyadh, Saudi Arabia

**Keywords:** chicken, *CXCLi1* gene, hemato-biochemistry, mRNA expression, salmonellosis

## Abstract

This study aimed to understand the differential and tissue-specific immune responses of three different chicken strains [Vanraja, Kashmir Commercial Layer (KCL), and broiler] by assessing the CXCLi1 chemokine mRNA expression in different tissues (liver, spleen, and cecum) in *Salmonella Typhimurium-*challenged birds. In addition, hematological and biochemical parameters were also assessed. *Salmonella enterica serovar Typhimurium* culture was used for induction of infection. Differential expression of the *CXCLi1* gene following induced infection was studied on different days post-infection (0, 1, 3, 5, 7, 9, 11, 13, and 15). An infection dose of 2 × 10^8^ CFU/mL produced the symptoms characteristics of salmonellosis. An immune response gene expression study revealed enhanced expression until the 5th–7th day post-infection, followed by a steady decrease until the 15th day post-infection. The overall gene expression was higher in broiler chicks than in KCL and Vanraja chicks. The tissue-specific response showed higher expression in the cecum followed by the spleen and liver. The real-time mRNA gene expression results indicated that commercial broilers are more susceptible than backyard chicks. Differential cellular responses revealed heterophilia and initial lymphopenia followed by lymphocytosis. Pronounced hemato-biochemical alterations were observed as the clinical indicators of *Salmonella* infection. These findings imply that the integration of disease-resistant alleles from indigenous or backyard poultry into high-performance exotic germplasm could improve *Salmonella* resistance in commercial poultry populations.

## 1 Introduction

*Salmonella enterica serovar Typhimurium* is a primary poultry pathogen that causes severe intestinal pathological variations in chickens. *Salmonellae* are facultative anaerobes, Gram-negative, non-spore-forming, and usually motile bacilli. The genus *Salmonella* comprises more than 2,500 closely related serotypes, which are mostly gastrointestinal pathogens affecting human and animals including poultry (Kim et al., 2006). *Salmonellae* are the leading cause of morbidity and mortality in poultry and result in significant economic losses (Bäumler et al., 1997; Prakash et al., 2005). These bacteria are known for their zoonotic potential and are a common cause of foodborne illnesses (Zajac et al., 2021). *Salmonella* continues to have a significant impact on global health and economy (Baptista et al., 2023). There were 41 major national outbreaks of salmonellosis in various poultry farms across India in the year 2014–15 that affected more than 43,740 birds, indicating the critical importance of salmonellosis in the poultry sector (Anonymous, 2015). Contamination of poultry products by *S. enterica serovar Typhimurium* is a major cause of foodborne infections and outbreaks (Bandaranayake et al., 2024). The ingested bacteria adhere to intestinal cells through fimbriae or pili and proliferate in the small intestine. They then penetrate enterocytes, where further multiplication occurs before they cross the lamina propria. They continue to proliferate, both freely and within macrophages. In young ones, the organisms are transported by macrophages to mesenteric lymph nodes. Furthermore, multiplication ultimately leads to septicemia, with localization of bacteria observed in many organs and tissues. The immune system is a host defense system comprising many biological structures and processes within an organism that protects it against disease. The cells of the immune system are special types of leukocytes, called lymphocytes, B and T cells. Cytokines, important proteins secreted by cells, play an essential role in immune and inflammatory responses. Chemokines are a class of cytokines that have chemoattractant activities that control the movement of immune cells (Kaiser and Staheli, 2008). The robust mucosal immune response in chicks following *Salmonella Typhimurium* challenge indicates activation of both humoral and cellular immune mechanisms, and thus it is recommended to consider administering a natural immune response stimulator at 1 day of age to enhance the chick’s ability to effectively combat infection and improve overall immunity (Mosa et al., 2024). The availability of avian genome sequences, along with the recent cloning of avian cytokines and chemokines, has led to a major shift in the ability to understand the host–pathogen interactions in avian hosts, particularly chickens (Hillier et al., 2004; Kaiser et al., 2009). A variety of inflammatory genes (IFNγ, IL8, IL10, INOS, MIP1β, TGFβ2, TLR4, and TLR15) have been temporally regulated (Bescucci et al., 2022). Chicken equivalent orthologs of INF-γ, IL-1β, IL-18, IL-10, IL-12, IL-17, IL-4, IL-13, IL-6, CCLi2, CXCLi1, and CXCLi2; transforming growth factors; tumor necrosis factors; and colony-stimulating factors have been cloned, sequenced, and identified (Kaiser, 2010). The elevations in levels of pro-inflammatory cytokines IL-6, IL-16, and IL-21 in the serum of *Salmonella Typhimurium-*challenged chicks suggest an active mucosal immune response to its infection. These cytokines play crucial roles in mediating inflammation and coordinating immune defense mechanisms at mucosal sites (Milby-Blackledge et al., 2024).

Plentiful approaches have been employed to fight salmonellosis in birds, but they have not proven to be highly effective and are found to suffer limitations. Because of huge pathogenic discovery, vaccination or antibiotic treatment is not always effectual (Alfaro et al., 2002). Vaccination alone cannot help in managing disease adequately, but it should be combined with disease resistance to exploit protection against diseases. Genetic selection for enhancing disease resistance in birds seems to be an encouraging approach (Gupta et al., 2010; Sivaraman and Kumar, 2010). Various selection strategies have been practiced for enhancing disease resistance in birds (Sarker et al., 2000; Pinard et al., 2004; Shivakumar and Kumar 2010; Shivakumar et al., 2011). Therefore, under the given scenario, there is a probably greater need to focus our attention to understand the genetics of diseases and immunity.

Consumption of poultry and poultry products has been on the rise in the Kashmir Valley of Jammu and Kashmir, India, over the last 10–12 years, leading to the development of abundant private poultry farms. However, rearing of birds on scientific lines is a major constraint with these farms. As a result, salmonellosis in human subjects poses an imminent threat. Therefore, in addition to biosecurity practices, breeding of disease-resistant chicken should be prioritized to optimize the concept of One World One Health One Medicine. Kashmir Commercial Layer (KCL) and Vanraja are two important chicken strains used in backyard poultry farming in the Kashmir Valley and are known for different morphological traits compared to broilers reared for commercial purposes. The superior adaptability of indigenous poultry to local environments coupled with their comparatively higher disease resistance than commercial broilers make them ideal candidates for elucidating disease mechanisms and host immune responses to pathogenic invasion. Insights gained from such studies could support the introgression of disease-resistant traits into high-yielding germplasm. Nevertheless, investigations into the expression profiles of backyard poultry vis-a-vis commercial broiler strains are still scarce.

## 2 Materials and methods

### 2.1 Experimental chicks

A total of 360 day-old chicks of Vanraja, KCL, and commercial broilers were purchased from the Center for Research on Poultry SKUAST-K, Srinagar, J&K, India; the Department of Animal Husbandry, Government of Jammu and Kashmir, India; and a reputed supplier, respectively, and reared in an experimental animal house at FVSc and AH, Shuhama, Srinagar. Chicks were acclimatized for 2 days and were maintained on a pre-starter ration for the first week and then on a starter ration for the remaining period. Birds were given *ad libitum* access to antibiotic-free feed and water.

### 2.2 Screening of chicks

To ensure that the chicks were free from *Salmonella* infection, fecal swabs were taken from the chicks and examined through bacteriological analysis for detecting the infected state. The swabs were inoculated in freshly prepared selenite broth, BGA, and MacConkey’s agar, and the growth was examined after 24–36 h of incubation at 37 °C. Based on culture and biochemical studies, chicks found *Salmonella-*negative were used for further studies.

### 2.3 Dose–response relationship study

*Salmonella enterica serovar Typhimurium* culture (KwikStik, LOT 180-171-1, REF 0180P, ATCC^R^51812^TM^) purchased from HIMEDIA Laboratories Pvt. Ltd. was used for studying the dose–response relationship and standardizing the inoculum required for inducing the infection. Confirmed *S. enterica serovar Typhimurium* colonies were inoculated in nutrient broth and kept in a shaking incubator at 37 °C overnight. Serial dilutions of 10^–1^, 10^–2^, 10^–3^, 10^–4^, 10^–5^, 10^–6^, 10^–7^, 10^–8^, 10^–9^, and 10^–10^ were made using PBS as the diluent. Then, 100 μL each of the 10^–5^, 10^–6^, 10^–7^, and 10^–8^ dilutions were spread on BGA plates and kept overnight in an incubator at 37 °C.

### 2.4 Experimental design, sampling, and bacteriological analysis

Chicks from each of three strains were divided into two experimental groups, an infected group and a control group, with 60 birds in each group. The infected group was exposed to the designated pathogen under controlled conditions, while the control group was maintained under similar environmental conditions without pathogen exposure. Sampling was carried out at different days post-infection (0, 1, 3, 5, 7, 9, 11, 13, and 15). The chicks (six controlled and six infected from each strain) were humanely sacrificed, and tissue samples were collected for further investigations. Liver, spleen, and cecum tissues were carefully excised and stored in sterile microcentrifuge tubes at −80 °C until RNA extraction. In addition, blood samples were collected from the chicks for hematological and biochemical analyses.

### 2.5 Extraction of total RNA and quantification of genes

RNA from the tissues was extracted using the TRIzol method. For removing trace amounts, DNase treatment was given using a Thermo Scientific DNase kit. cDNA synthesis was conducted using Thermo Scientific RevertAid First-Strand cDNA Synthesis Kit with oligo dT primers. The primers employed for gene amplification were previously described by Setta et al., 2012; He et al., 2013, and the details of these primers are given in Table 1. The PCR validation of cDNA was conducted by PCR amplification under standard conditions, and the amplified products were run on 2.5% agarose gel. The amplified products of the *β actin* (160 bp) and *CXCLi1* (119 bp) genes were obtained in different strains of poultry. The SYBR Green I assay was used for quantification of the gene of interest and detection of amplified products. A pre-formulated real-time master mix containing buffer, dNTPs, DNA polymerase, and SYBR Green I dye was used. The mRNA quantification of the genes was carried out in Roche LightCycler 480 II and was determined using the 2^−ΔΔCT^ method (Livak and Schmittgen, 2001), where ΔΔCT corresponds to the difference between the CT measured for the mRNA level of each tissue and the CT measured for the mRNA level of the reference gene, ΔCT = CT_(target gene)_ – mean CT_(*β*-Actin)_ and CT_(CXCLi1)_.

**TABLE 1:** Oligonucleotide primer sequences used in real-time PCR for the gene expression study.

Target gene	Primer set 5′–3′		Product size (bp)	Annealing temperature (^o^C)	References
CXCLi1	F	CCA​GTG​CAT​AGA​GAC​TCA​TTC​CAA​A	119	60	Setta et al. (2012)
	R	TGC​CAT​CTT​TCA​GAG​TAG​CTA​TGA​CT			
β actin	F	TGGCATTGCTGACAGGAT	160	63	He et al. (2013)
	R	CTGCTTGCTGATCCACAT			

F, forward; R, reverse.

### 2.6 Hemato-biochemical analysis

Hematological parameters like WBCs and lymphocytes were estimated by using a hematological analyzer (Melet Schloesing Laboratories MS4S). Serum biochemical parameters like total serum protein and albumin were determined using a semi-automated analyzer (Photometer 5010V5+, Robert Riele Germany) with commercial kits.

### 2.7 Statistical analysis

The resulting data were analyzed using a three-factorial experimental design in R software to evaluate the main and interactive effects of strain, treatment, and time post-infection on gene expression levels. Analysis of variance was performed to determine statistically significant differences among the factors. The comparative Ct method, also known as the ΔΔCt method, was used to determine the relative quantification of gene expression. Using the 2^−ΔΔCt^ method, the data were expressed as fold change in target gene expression, normalized to an endogenous reference gene and relative to the calibrator sample.

## 3 Results

### 3.1 *Salmonella Typhimurium* strain confirmation from sacrificed birds

The contents from the cecum of infected sacrificed chickens were inspected for *Salmonella* organisms using Brilliant Green Agar at 37 °C for 18–24 h for confirmation of induced infection. Samples were also subjected to biochemical and morphological tests and Gram’s staining. After streaking, pink and pale white colonies were produced on BGA and MacConkey Agar, respectively. The IMViC test showed that the isolates were negative for indole and Voges–Proskauer tests and positive for methyl red and citrate utilization tests. Pink Gram-negative rods were obtained by Gram staining. The isolates were also urease-positive.

### 3.2 Study of the dose–response relationship

Doses of 2 × 10^7^, 2 × 10^8^, and 1 × 10^9^ CFU/mL were administered to the chicks orally, and they were observed for symptoms of salmonellosis. In the latter group (1 × 10^9^ CFU/mL), considerable mortality was observed, rendering the dose unsuitable for use in the study. However, the dose of 2 × 10^8^ CFU/mL produced the maximum symptoms characteristic of salmonellosis, like dullness, marked depression, progressive weakness, closed eyes, reluctance to move, inappetence, increased thirst, ruffled feathers, drooping of wings, lowering of the head and diarrhea, without incurring unbearable mortality. Hence, this dose was used for induction of infection in the experimental chicks. Postmortem examination revealed intestinal hemorrhages, bronze discoloration of the liver, elevated white nodular lesions on the cardiac ventricles, and prominent necrotic foci on the liver. All these signs and symptoms confirm *Salmonella Typhimurium* infection.

### 3.3 Effect of *Salmonella Typhimurium-*induced infection on *CXCLi1* gene expression at different days post-infection

The mRNA expression of the *CXCLi1* gene at days 0, 1, 3, 5, 7, 9, 11, 13, and 15 day post-infection was studied in the infected and control groups of experimental animals.

#### 3.3.1 *CXCLi1* gene expression in the liver at different days post-infection

**FIGURE 1:**
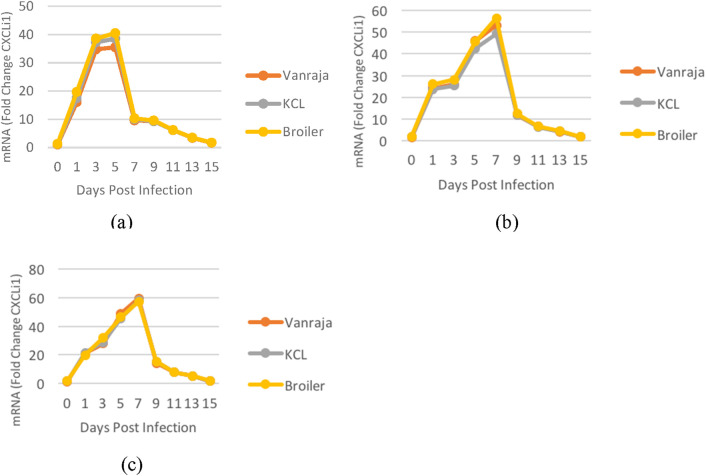
Graphical representation of the fold change expression of the *CXCLi1* gene calculated using the ΔΔC_T_ method in the **(a)** liver; **(b)** spleen; **(c)** cecum of Vanraja, KCL, and broiler chicks at different days post-infection.

The mRNA expression levels in the liver of Vanraja chicks showed an increase of 1.09- to 35.51-fold at 0–5 days post-infection and then decreased (from 9.65- to 1.74-fold). The mRNA expression levels in the liver of KCL chicks increased from day 0 to day 5 (1.21- to 38.58-fold) and thereafter decreased from 10.06- to 1.76-fold. The mRNA expression levels in the liver of broiler chicks changed from 1.29- to 40.50-fold at 0–5 days post-infection and thereafter decreased to 1.77-fold only on the 15th day post-infection (Figure 1a).

#### 3.3.2 *CXCLi1* gene expression in the spleen at different days post-infection

*CXCLi1* gene mRNA fold expression in the spleen of Vanraja chicks increased significantly from days 0 to 7, increasing from 1.37- to 53.08-fold, and decreased thereafter (11.63- to 1.72-fold). With respect to the spleen, the mRNA fold expression of the *CXCLi1* gene in KCL chicks increased during the first week of infection from 1.75- to 49.18-fold and decreased from day 7 onward (from 11.96 to 1.68 fold). Moreover, *CXCLi1* gene mRNA fold expression in the spleen of broiler chicks increased during the first week post-infection from 1.87- to 56.49-fold and decreased thereafter (from 12.38- to 1.75-fold) (Figure 1b).

#### 3.3.3 *CXCLi1* gene expression in the cecum at different days post-infection

With reference to the *β* actin gene, the expression of *CXCLi1* increased significantly at days 0 to 7 (1.25- to 59.56-fold) in the cecum of Vanraja chicks. However, from day 7 onward, it decreased from 14.22- and reached 1.55-fold at day 15 post-infection. The mRNA fold expression of the *CXCLi1* gene in the cecum of KCL chicks increased up to the end of the first week post-infection from 1.58- to 58.08-fold, but decreased from the second week onward (from 15.24- to 1.85-fold). The mRNA fold expression of the *CXCLi1* gene in the cecum of broiler chicks also increased from days 0 to 7 (1.97- to 57.28-fold) and then decreased from the second week onward (15.45- to 1.79-fold) (Figure 1c).

#### 3.3.4 Overall mRNA expression levels of the *CXCLi1* gene in different tissues of experimental birds

The overall fold expression levels of the *CXCLi1* gene in the infected liver in Vanraja, KCL, and broiler chicks were 13.086 ± 1.729, 13.889 ± 1.882, and 14.612 ± 1.971, respectively, and differed significantly (p < 0.05). The fold expression levels in the spleen were also statistically significant (19.401 ± 2.507, 18.500 ± 2.315, and 20.351 ± 2.604, respectively). The cecal fold expression levels were 20.791 ± 2.730, 20.996 ± 2.565, and 20.868 ± 2.616 in Vanraja, KCL, and broiler chicks, respectively, and were statistically non-significant. The overall fold expression levels of the *CXCLi1* gene in the liver, spleen, and cecum of infected chicks of different strains were 13.862 ± 1.070, 19.417 ± 1.423, and 20.885 ± 1.514, respectively, and differed significantly (p < 0.05), indicating higher expression in the cecum followed by the spleen and liver. On different days (0, 1, 3, 5, 7, 9, 11, 13, and 15) post-infection, the overall expression levels were 1.717 ± 0.231, 21.023 ± 0.444, 30.919 ± 0.689, 43.239 ± 0.604, 40.413 ± 2.983, 12.100 ± 0.341, 6.813 ± 0.106, 4.535 ± 0.140, and 1.734 ± 0.020, respectively, indicating a significant increase until the fifth DPI, followed by a significant decrease until the 15th DPI. The expression levels on day 15 were comparable to those on day 1 post-infection. The overall fold expression levels of the *CXCLi1* gene were 17.759 ± 1.380, 17.795 ± 1.324, and 18.610 ± 1.404 in Vanraja, KCL, and broiler chicks, respectively; the expression level was significantly different (p < 0.05) in broiler chicks compared to that in Vanraja and KCL chicks (Table 2).

**TABLE 2:** CXCLi1 gene expression (fold change) in *Salmonella Typhimurium-*induced infection at different days post-infection.

Tissue	Liver			Sub mean	Spleen			Sub mean	Cecum			Sub mean	Factor mean	
Strain DPI	Vanraja	KCL	Broiler		Vanraja	KCL	Broiler		Vanraja	KCL	Broiler		Days	Strain
0	1.09	1.21	1.29	1.197	1.37	1.75	1.87	1.663	1.25	1.58	1.97	1.600	1.717^h^ ± 0.231	17.759^B^ ± 1.380
1	16.11	17.50	19.70	17.770	24.25	23.59	25.99	24.610	20.82	21.41	19.84	20.690	21.023^d^ ± 0.444	17.795^B^ ± 1.324
3	34.77	37.27	38.59	36.877	25.81	25.28	28.05	26.380	27.86	28.64	32.00	29.500	30.919^c^ ± 0.689	18.610^A^ ± 1.404
5	35.51	38.58	40.50	38.197	45.89	42.52	45.57	44.660	48.84	45.57	46.20	46.870	43.239^a^ ± 0.604	​
7	9.65	10.06	10.34	10.017	53.08	49.18	56.49	52.917	59.56	58.08	57.28	58.307	40.413^b^ ± 2.983	​
9	9.32	9.32	9.58	9.407	11.63	11.96	12.38	11.990	14.22	15.24	15.45	14.970	12.100^e^ ± 0.341	​
11	6.15	6.15	6.23	6.177	6.63	6.19	6.59	6.470	7.67	7.89	7.89	7.817	6.813^f^ ± 0.106	​
13	3.43	3.48	3.51	3.473	4.23	4.35	4.47	4.350	5.35	5.39	5.39	5.377	4.535^g^ ± 0.140	​
15	1.74	1.76	1.77	1.757	1.72	1.68	1.75	1.717	1.55	1.65	1.79	1.663	1.734^h^ ± 0.020	​
Overall mean	13.086^m^ ± 1.729	13.889^n^ ± 1.882	14.612^o^ ± 1.971	13.862^X^ ± 1.070	19.401^j^ ± 2.507	18.500^k^ ± 2.315	20.351^l^ ± 2.604	19.417^Y^ ± 1.423	20.791^a^ ± 2.730	20.996^a^ ± 2.565	20.868^a^ ± 2.616	20.885^Z^ ± 1.514	​	​

Means with different superscripts are significantly different (p < 0.05). Capital letters (X, Y & Z) in superscripts indicate significant overall mean differences among tissues irrespective of chicken strain. Lowercase letters in superscripts indicate significant overall differences among different tissues (liver, spleen and caecum) of different chicken strains.

CD (p < 0.05); Tissue = 0.291; Strain = 0.291; Day = 0.505.

Tissue × Strain = 0.505; Tissue × Day = 0.875; Strain × Day = 0.875; Tissue × Strain × Day = 1.516.

### 3.4 Effect of induced infection on hematological and biochemical parameters

#### 3.4.1 Heterophil count (10^3^/μL)

**FIGURE 2:**
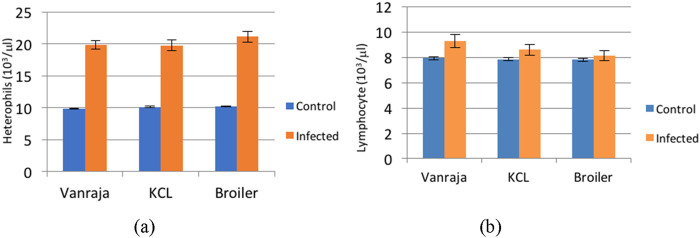
Effect of *Salmonella Typhimurium-*induced infection on **(a)** heterophils (10^3^/μL) and **(b)** lymphocytes (10^3^/μL) in control and infected groups in three different strains.

The mean values showed an increasing trend in infected groups until the 7th DPI. Thereafter, it started to decrease in infected groups. A continuous decrease in control groups was observed throughout the study. Heterophil counts differed significantly between infected and control groups in all the three strains. In control groups, the counts were 9.853 ± 0.100, 10.138 ± 0.0.093, and 10.241 ± 0.105 in Vanraja, KCL, and broiler chicks, respectively, and there were no significant differences observed between the control groups. A significant difference was observed in infected groups, with higher counts in broilers (21.175 ± 0.846) than in KCL (19.781 ± 0.866) and Vanraja (19.916 ± 0.661) chicks (p < 0.05). The overall mean values of the heterophil count in the control and infected groups were 10.075 ± 0.058 and 20.290 ± 0.459, respectively, and were found statistically significant (p < 0.05) (Table 3; Figure 2a).

**TABLE 3:** Effect of *Salmonella Typhimurium-*induced infection on the heterophil count (10^3^/μL) at different days post-infection.

Strain DPI	Control			Sub mean	Infected			Sub mean	Factor mean	
	Vanraja	KCL	Broiler		Vanraja	KCL	Broiler		Days	Strain
0	10.682	10.624	10.412	10.573	10.664	10.668	10.398	10.577	10.575^f^ ± 0.151	14.884^B^ ± 0.589
1	10.493	10.581	10.398	10.491	16.413	15.842	14.911	15.722	13.106^e^ ± 0.469	14.959^B^ ± 0.636
3	10.168	10.554	10.381	10.368	20.680	18.998	18.611	19.763	15.065^e^ ± 0.753	15.708^A^ ± 0.677
5	9.864	10.234	10.311	10.136	26.415	22.681	26.842	26.979	18.558^a^ ± 1.445	​
7	9.721	10.084	10.308	10.038	25.410	23.961	28.212	27.161	18.599^a^ ± 1.473	​
9	9.621	9.984	10.274	9.960	23.426	25.802	28.042	25.370	17.665^b^ ± 1.344	​
11	9.448	9.904	10.271	9.874	21.412	22.532	24.680	22.851	16.363^c^ ± 1.126	​
13	9.394	9.680	9.987	9.687	18.610	19.685	20.420	19.624	14.656^d^ ± 0.867	​
15	9.241	9.593	9.823	9.552	16.214	17.831	18.4620	17.569	13.561^e^ ± 0.699	​
Overall mean	9.853^x^ ± 0.100	10.138^x^ ± 0.093	10.241^x^ ± 0.105	10.075^X^ ± 0.058	19.916^y^ ± 0.661	19.781^y^ ± 0.866	21.175^z^ ± 0.846	20.290^Y^ ± 0.459	​	​

Means with different superscripts are significantly different (p < 0.05). Capital letters X and Y in superscripts indicate significant overall mean differences in heterophil counts between uninfected and infected groups irrespective of chicken strain. Lowercase letters in superscripts indicate overall mean differences in heterophil counts among different chicken strains within the uninfected groups and within the infected groups.

CD (p < 0.05); Days = 0.578; Strain = 0.334; Treatment = 0.272.

Days × Strain = 1.002; Days × Treatment = 0.818; Strain × Treatment = 0.472; Days × Strain × Treatment = 1.417.

#### 3.4.2 Lymphocyte count (10^3^/μL)

There was a decrease in the lymphocyte count in infected groups until the 5th DPI. This initial lymphopenia was followed by lymphocytosis in the infected groups. The overall lymphocyte count in the control groups increased gradually throughout the experimental study. Mean lymphocyte values in all the three strains differed significantly between infected and control groups. With respect to the control groups, the mean values were 7.935 ± 0.103, 7.862 ± 0.107, and 7.811 ± 0.105 in Vanraja, KCL, and broiler chicks, respectively, and were found non-significant. However, the mean values differed significantly in the infected groups, with higher counts in Vanraja (9.323 ± 0.519) than in KCL (8.609 ± 0.432) and broiler (8.135 ± 0.383) chicks (p < 0.05). The overall lymphocyte counts irrespective of the strain in the control and infected groups were 7.871 ± 0.060 and 8.689 ± 0.260, respectively, and were statistically significant (p < 0.05) (Table 4; Figure 2b).

**TABLE 4:** Effect of *Salmonella Typhimurium* infection on the lymphocyte count (10^3^/μL) at different days post-infection.

Strain DPI	Control			Sub mean	Infected			Sub mean	Factor mean	
	Vanraja	KCL	Broiler		Vanraja	KCL	Broiler		Days	Strain
0	7.005	7.083	7.076	7.055	6.989	7.088	7.068	7.048	7.052^ef^ ± 0.066	8.629^A^ ± 0.272
1	7.239	7.112	7.121	7.157	6.732	6.912	6.868	6.837	6.997^efg^ ± 0.056	8.235^B^ ± 0.224
3	7.428	7.216	7.136	7.260	6.120	6.628	6.542	6.430	6.845^fg^ ± 0.096	7.973^C^ ± 0.198
5	7.648	7.593	7.329	7.523	5.801	6.111	6.208	6.040	6.782^g^ ± 0.143	​
7	7.812	7.868	7.842	7.841	6.916	6.081	6.216	6.404	7.123^e^ ± 0.146	​
9	8.044	7.968	8.006	8.006	8.924	7.146	6.842	7.637	7.822^d^ ± 0.138	​
11	8.426	8.340	8.312	8.359	11.031	9.326	7.931	9.429	8.894^c^ ± 0.186	​
13	8.641	8.594	8.642	8.626	14.816	12.864	10.682	12.787	10.707^b^ ± 0.414	​
15	9.168	8.987	8.831	8.995	16.581	15.329	14.858	15.589	12.292^a^ ± 0.583	​
Overall mean	7.935^x^ ± 0.103	7.862^x^ ± 0.107	7.811^x^ ± 0.105	7.871^X^ ± 0.060	9.323^w^ ± 0.519	8.609^y^ ± 0.432	8.135^z^ ± 0.383	8.689^Y^ ± 0.260	​	​

Means with different superscripts are significantly different (p < 0.05). Capital letters X and Y in superscripts indicate significant overall mean differences in lymphocyte counts between uninfected and infected groups irrespective of chicken strain. Lowercase letters in superscripts indicate overall mean differences in lymphocyte counts among different chicken strains within the uninfected groups and within the infected groups.

CD (p < 0.05); Days = 0.242; Strain = 0.140; Treatment = 0.114.

Days × Strain = 0.420; Days × Treatment = 0.343; Strain × Treatment = 0.198; Days × Strain × Treatment = 0.594.

#### 3.4.3 Serum albumin (g/dL)

**FIGURE 3:**
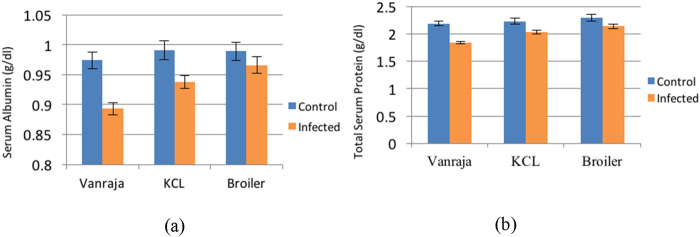
Effect of *Salmonella Typhimurium-*induced infection on **(a)** serum albumin (g/dL) and **(b)** total serum protein (g/dL) in control and infected groups in three different strains.

There was a marked increase in the serum albumin values in infected and control groups throughout the study. Mean serum albumin values differed significantly between infected and control groups. The overall mean values of serum albumin in the control groups of Vanraja, KCL, and broiler chicks were 0.974 ± 0.014, 0.991 ± 0.016, and 0.989 ± 0.015, respectively, and were statistically non-significant. In the infected groups, the mean values were 0.893 ± 0.010, 0.938 ± 0.011, and 0.966 ± 0.014, respectively, and were found statistically significant, with higher counts in broiler chicks (p < 0.05). The overall mean AST values in the control and infected groups were 0.985 ± 0.008 and 0.932 ± 0.007, respectively, and were found statistically significant (p < 0.05) (Table 5; Figure 3a).

**TABLE 5:** Effect of *Salmonella Typhimurium* infection on serum albumin (g/dL) at different days post-infection.

Strain DPI	Control			Sub mean	Infected			Sub mean	Factor mean	
	Vanraja	KCL	Broiler		Vanraja	KCL	Broiler		Days	Strain
0	0.864	0.884	0.880	0.876	0.861	0.891	0.903	0.885	0.881^f^ ± 0.013	0.933^B^ ± 0.009
1	0.891	0.913	0.908	0.904	0.862	0.901	0.904	0.889	0.897^f^ ± 0.011	0.964^A^ ± 0.009
3	0.912	0.924	0.931	0.922	0.865	0.913	0.918	0.899	0.911^ef^ ± 0.009	0.978^A^ ± 0.010
5	0.934	0.938	0.946	0.939	0.869	0.921	0.928	0.906	0.923^ef^ ± 0.014	​
7	0.974	0.988	0.973	0.978	0.874	0.924	0.941	0.913	0.946^de^ ± 0.012	​
9	0.994	1.032	0.994	1.007	0.894	0.963	0.968	0.942	0.974^cd^ ± 0.013	​
11	1.014	1.061	1.084	1.053	0.921	0.969	1.004	0.965	1.009^bc^ ± 0.014	​
13	1.054	1.074	1.086	1.071	0.930	0.973	1.040	0.981	1.026^ab^ ± 0.018	​
15	1.132	1.103	1.103	1.113	0.964	0.989	1.085	1.013	1.063^a^ ± 0.019	​
Overall mean	0.974^x^ ± 0.014	0.991^x^ ± 0.016	0.989^x^ ± 0.015	0.985^X^ ± 0.008	0.893^w^ ± 0.010	0.938^y^ ± 0.011	0.966^z^ ± 0.014	0.932^Y^ ± 0.007	​	​

Means with different superscripts are significantly different (p < 0.05). Capital letters X and Y in superscripts indicate significant overall mean differences in serum albumin values between uninfected and infected groups irrespective of chicken strain. Lowercase letters in superscripts indicate overall mean differences in serum albumin values among different chicken strains within the uninfected groups and within the infected groups.

CD (p < 0.05); Days = 0.038; Strain = 0.023; Treatment = 0.018.

Days × Strain = 0.067; Days × Treatment = 0.054; Strain × Treatment = 0.032; Days × Strain × Treatment = 0.095.

#### 3.4.4 Total serum proteins (g/dL)

There was a continuous increase in the serum protein levels in infected and control groups throughout the experiment. Overall mean values differed significantly between infected and control groups in all the three strains. The mean values of total protein between three different control groups were 2.190 ± 0.042, 2.234 ± 0.052, and 2.298 ± 0.057 in Vanraja, KCL, and broiler chicks, respectively, and there was no significant difference observed between these groups. However, in the infected groups, the mean values were 1.842 ± 0.019, 2.036 ± 0.034, and 2.139 ± 0.043, with higher values in broilers, and significant differences were observed between all the three strains in the infected groups. The overall protein levels in the control and infected groups were 2.207 ± 0.030 and 2.006 ± 0.020, respectively, and were found statistically significant (p < 0.05) (Table 6; Figure 3b).

**TABLE 6:** Effect of *Salmonella Typhimurium* infection on total serum proteins (g/dL) at different days post-infection.

Strain DPI	Control			Sub mean	Infected			Sub mean	Factor mean	
	Vanraja	KCL	Broiler		Vanraja	KCL	Broiler		Days	Strain
0	1.714	1.746	1.802	1.754	1.710	1.751	1.813	1.758	1.756^g^ ± 0.016	1.966^A^ ± 0.026
1	1.771	1.840	1.864	1.825	1.746	1.796	1.823	1.788	1.807^gh^ ± 0.015	2.135^B^ ± 0.032
3	1.886	1.913	1.891	1.897	1.773	1.811	1.868	1.817	1.857^g^ ± 0.018	2.218^C^ ± 0.036
5	1.990	2.085	2.084	2.053	1.814	1.987	1.987	1.929	1.991^f^ ± 0.033	​
7	2.103	2.213	2.314	2.210	1.830	2.042	2.123	1.998	2.104^e^ ± 0.036	​
9	2.168	2.311	2.486	2.322	1.856	2.113	2.241	2.070	2.196^d^ ± 0.040	​
11	2.214	2.540	2.643	2.466	1.921	2.168	2.310	2.133	2.299^c^ ± 0.051	​
13	2.354	2.631	2.714	2.566	1.941	2.234	2.423	2.199	2.383^b^ ± 0.052	​
15	2.610	2.831	2.883	2.775	1.990	2.421	2.663	2.358	2.566^a^ ± 0.058	​
Overall mean	2.190^x^ ± 0.042	2.234^x^ ± 0.052	2.298^x^ ± 0.057	2.207^X^ ± 0.030	1.842^w^ ± 0.019	2.036^y^ ± 0.034	2.139^z^ ± 0.043	2.006^Y^ ± 0.020	​	​

Means with different superscripts are significantly different (p < 0.05). Capital letters X and Y in superscripts indicate significant overall mean differences in total serum protein values between uninfected and infected groups irrespective of chicken strain. Lowercase letters in superscripts indicate overall mean differences in total serum protein values among different chicken strains within the uninfected groups and within the infected groups.

CD (p < 0.05); Days = 0.074; Strain = 0.043; Treatment = 0.035.

Days × Strain = 0.129; Days × Treatment = 0.106; Strain × Treatment = 0.061; Days × Strain × Treatment = 0.183.

## 4 Discussion

In this study, administration of an infectious dose of $2\times 10^{8}$ CFU/mL resulted in the development of clinical signs and symptoms typical of salmonellosis in chicks. Among the inoculation routes tested, the oral route was found to be the most effective for establishing infection, consistent with previous findings (Cox et al., 1973). Upon postmortem examination, *Salmonella Typhimurium-*infected chicks were found to have necrotic and hemorrhagic foci in the liver, elevated white nodular lesions on the ventricles, bronze discoloration of the liver, fibrinous exudates in the pericardial sac, congestion of the intestines and lungs, and inflamed ceca containing cheesy necrotic debris. These findings are well-supported by those of other studies in which necrotic and hemorrhagic foci have been found on the liver in *Salmonella-*infected chicken (Chisti et al., 1985). Infection of chickens with *S. enterica* induced metabolic disturbances, leading to oxidative stress and heightened inflammation. In addition, there was an increase in biomarkers associated with hepatocellular carcinoma, indicating potential progression toward liver malignancy (Lu et al., 2024).

Real-time expression analyses were carried out at multiple time points post-infection and in different tissues, namely, the liver, spleen, and cecum. The *CXCLi1* mRNA expression levels increased significantly until the 5th–7th day post-infection, after which a gradual decrease was observed through the 15th DPI. The expression was higher in the cecum than in the spleen and liver. Histopathologic changes were also observed in the small and large intestines, including the colon of chicks inoculated with *Salmonella Typhimurium*, which increased in magnitude over the experimental time period (Bescucci et al., 2022). Expression levels were also influenced by strain, with higher levels observed in broiler chicks than in the other two lines. Increased expressions of immune response genes until the 7th–9th DPI could be attributed to elicitation of cellular immune response/acquired immunity phase of infection for early clearance of the *Salmonella* infection (Caron et al., 2002). The higher fold increase in the immune response gene expression in the cecum could be related to increased *Salmonella* colonization and multiplication in the cecum compared to the spleen and liver. The key enzyme levels for arachidonic acid production and metabolism (phospholipase A2 PLA2 and cyclooxygenase-2 COX-2) in chicken cecum tissues were increased after *Salmonella Typhimurium* infection (Chen et al., 2025). Immune response gene expression in the spleen responsible for early clearance of the pathogen could be due to the large volume of macrophages present in the spleen (Hassanin, 2011). Immune response gene expression in the liver responsible for bacterial clearance could be attributed to the late-stage (migration of bacteria through blood to the liver) multiplication of *Salmonella* in the liver (Barrow et al., 2000). The higher expression levels of immune response genes observed in broiler chicks compared to KCL and Vanraja strains indicated higher colonization and invasion of *Salmonella Typhimurium* in broilers. This suggests that the backyard poultry strains (KCL and Vanraja) exhibit relatively higher resistance to *Salmonella Typhimurium* infection compared to the commercial broiler strain. The enhanced resistance observed in indigenous or backyard strains may be attributed to their well-adapted immune mechanisms and genetic resilience, which have evolved through long-term exposure to diverse environmental conditions and a wide range of pathogens. Unlike commercial broiler strains that are selectively bred primarily for rapid growth and production traits, backyard strains are often naturally selected for survival and disease tolerance. This continuous exposure to environmental stressors and microbial challenges likely contributes to the development of a more robust and responsive immune system, enabling these strains to mount a more effective defense against *Salmonella Typhimurium* infection. This underscores the importance of genetic factors in shaping immune gene expression profiles during *Salmonella* infection, which could have implications for breeding strategies aimed at enhancing disease resistance (Michael et al., 2022). Strain influence could also be due to differences in the efficiency of the phagocytic system that restricts bacterial growth during the initial infection phase (Bumstead and Barrow, 1993). Cheeseman et al. (2007) observed the influence of strain genetics on cytokine mRNA expression in young chickens, potentially explaining some generalized immune response differences between strains. Cheeseman et al. (2008) observed upregulation of CXCLi1 and CXCLi2 expression and macrophage cell populations in the ceca of *Salmonella enteritidis-*infected young chicken. Using 16s rDNA sequencing, the relative mRNA expression levels of inflammatory factors in cecal flora were increased after infection with *Salmonella Typhimurium*, including interferon-γ (IFN-γ), transforming growth factor-β1 (TGF-β1), interleukin-4 (IL-4), and interleukin-6 (IL-6) (Chen et al., 2025). Beal et al. (2004) and Berndt et al. (2007) observed upregulated expressions of IL-1β, IL-6, IL-17, IL-22, and IFNγ, together with iNOS in the cecum of infected chickens. He et al. (2013) observed significantly increased splenic NRAMP1 mRNA expression from days 0 to 5 post-infection, followed by a significant decrease from day 7 onward. Setta et al. (2012) observed upregulated expressions of CXCLi1 and CXCLi2 genes in the cecal tonsils of newly hatched chickens infected with *S. enteritidis* compared to that of lipopolysaccharide-induced tumor necrosis factor alpha factor (LITAF) in *Salmonella gallinarum-*infected birds. Braukmann et al. (2015) observed elevated mRNA expressions of inducible nitric oxide synthase, IL-12, IL-18, and LITAF following infection of primary avian splenic macrophages with *S. enterica serovar Typhimurium* and *Salmonella infantis*. It has also been observed that both IL-12 and IL-17 levels were significantly increased at 6 h post-infection (p < 0.05) in the cecal tonsil of chickens challenged with *Eimeria tenella* and hence indicates an early immune response involving these cytokines in the local lymphoid tissue following infection (Wang et al., 2024).

Dangana et al. (2010) observed a significant WBC and lymphocyte count with the reticulocyte count being significantly higher and a relatively higher neutrophil count in *Salmonella Typhimurium* and *para-Typhimurium* in humans compared to apparently healthy control individuals. In addition, there was a significant increase in monocyte and eosinophil count, but no significant difference was observed in basophil counts. The overall mean value of WBC and heterophil count increased significantly (P < 0.05) from day 1 post-infection to day 7 in infected groups compared to respective controls. There was also initially a significant decrease (P < 0.05) in the overall mean of the lymphocyte count from day 1 to day 5 post-infection. The initial lymphopenia was followed by lymphocytosis in experimental animals throughout the experimental period. A significant increase (P < 0.05) in total white blood cell and heterophil counts observed post-infection was consistent with that reported by Berchieri (2000), who attributed the increase in the leukocyte count to rapid multiplication of *S. gallinarum* inside the phagocytes, with subsequent cell lysis and release of the bacterium into the extracellular compartment evoking a strong immune response. The increase in the heterophil count in the *Salmonella-*infected chicken is because heterophils respond most in bacterial infection (Feldmann et al., 2000). The leukocytosis observed in this study coincided with the period of manifestation of the clinical signs (depression, somnolence, anorexia, ruffled feathers, and greenish-to-yellowish diarrhea) of fowl typhoid in the infected birds. This finding conformed with the reports of Berchieri (2000) and Freitas Neto et al. (2007). In addition, the possible bacterial invasion of the target organs, such as the liver, spleen, kidneys, and ovarian follicle, might cause an increase in peripheral blood leukocytes as an inflammatory response. Leukocytosis due to relative heterophilia early in response to the *Salmonella* challenge could be attributed to the role of heterophils in natural immunity and cellular defense against microbial infections, along with the response to acute inflammatory and degenerative/necrotic changes in internal organs and to bone marrow hyperplasia (Assoku and Penhale, 1970). Their migration to inflammatory sites could also be a reason for their decrease in the blood level at later time points (Harmon, 1998). During invasive *Salmonella* infection, PAMPs and DAMPs triggered the innate immune system, leading to activation and recruitment of neutrophils and macrophages and the production of pro-inflammatory cytokines (Mogensen, 2009). The lymphopenia observed post-infection in the infected chicks might be due to stress of infection with *Salmonella,* inducing the adrenal gland’s release of cortical hormones that destroy the lymphocytes (DeGroot and Morris, 1950). In similar studies by Shah et al. (2013) in poultry, it was found that WBC, heterophil, and lymphocyte counts in the infected group increased significantly (p < 0.05) as compared to the control groups. The findings are in agreement with the observations made by Madhuri and Sadana (2000), Kokosharov (2000), and Shah et al. (2013), who noticed a significant (P < 0.05) increase in WBC, heterophil, and lymphocyte counts in infected groups compared to their respective controls. Xie et al. (2003) observed infection/induced elevation in lymphocyte concentration and related to the adaptive immunity.

A significant (p < 0.05) decrease in serum total protein and albumin count on different days post-infection in the infected chicks compared to their controls could be attributed to severe liver damage, resulting in malfunctioning of the liver and decreased protein synthesis (Grant and Tietz, 1987). A decrease in the total protein count could also be related to kidney damage, leading to protein loss, or due to proteolysis caused by *Salmonella* infection (Kokosharov, 2000). A decrease in the serum albumin concentration could be due to an acute phase response invoked/generated due to induced infection. As a negative acute-phase protein, albumin concentration decreases during the acute-phase response (Gruys et al., 2005). Similar results have been reported in infected broiler chicks (Shah et al., 2013). However, they are in complete disagreement with our findings. The increased blood protein concentration at all time points in induced salmonellosis has been associated with altered production of proteins related to the acute-phase response (Kancko, 1989; Aldred and Schreiber, 1993).

## 5 Conclusion

In conclusion, the expression profiling indicated that broilers were more susceptible to *S. enterica serovar Typhimurium* infection, whereas the Vanraja strain exhibited higher resistance. Tissue-specific gene expression analysis revealed the highest fold changes in the cecum, followed by the spleen and liver. Temporal expression analysis showed peak mRNA expression levels between the 5th and 7th day post-infection. Significant hemato-biochemical alterations served as clinical indicators for the establishment of *Salmonella* infection. These findings suggest that introgression of disease-resistant genes from indigenous or backyard poultry into high-yielding exotic germplasm could enhance resistance to *Salmonella* infection in commercial poultry populations.

## Data Availability

The original contributions presented in the study are included in the article/supplementary material; further inquiries can be directed to the corresponding authors.
